# Exploring the Feasibility of Utilizing Limited Gene Panel Circulating Tumor DNA Clearance as a Biomarker in Patients With Locally Advanced Non-Small Cell Lung Cancer

**DOI:** 10.3389/fonc.2022.856132

**Published:** 2022-03-28

**Authors:** Brendan Knapp, Laura Mezquita, Siddhartha Devarakonda, Mihaela Aldea, Saiama N. Waqar, Kym Pepin, Jeffrey P. Ward, Angela Botticella, Karen Howarth, Charlene Knape, Clive Morris, Ramaswamy Govindan, Benjamin Besse, Daniel Morgensztern

**Affiliations:** ^1^Department of Medicine, Division of General Medicine, Washington University School of Medicine, St. Louis, MO, United States; ^2^Medical Oncology Department, Gustave Roussy Cancer Campus, Villejuif, France; ^3^Department of Medicine, Division of Oncology, Washington University School of Medicine, St. Louis, MO, United States; ^4^Radiation Oncology Department, Gustave Roussy Cancer Campus, Villejuif, France; ^5^Department of Clinical Genomics, Inivata Limited, Cambridge, United Kingdom; ^6^Inivata Inc, Research Triangle Park, Durham, NC, United States

**Keywords:** ctNDA, fixed gene panel, prognostication, non-small cell lung cancer, commercially available

## Abstract

**Introduction:**

Circulating tumor DNA (ctDNA) testing may identify patients at high risk for recurrence following chemoradiation (CRT) for locally advanced non-small cell lung cancer (LA-NSCLC). We evaluated the feasibility of ctDNA testing on a readily available commercial fixed-gene panel to predict outcomes in patients with LA-NSCLC.

**Methods:**

Plasma of 43 patients was collected at CRT initiation (pre-CRT), completion (post-CRT1), quarterly follow up for 12 months (post-CRT2, 3, 4, 5 respectively) after CRT, and at disease progression. ctDNA analysis was performed using InVisionFirst^®^-Lung to detect mutations in 36 cancer-related genes. ctDNA clearance was defined as absence of pre-CRT variants at post-CRT1. Patients without detectable pre-CRT variants or no post-CRT1 samples were excluded.

**Results:**

Twenty eight of 43 patients (65%) had detectable variants pre-CRT. Nineteen of 43 patients (44%) had detectable pre-CRT variants and post-CRT1 samples and were included in analysis. Median age at diagnosis was 65 years (43-82), and most patients had stage IIIB disease (10/19, 53%). Two patients died from non-cancer related causes before post-CRT2 and were excluded from further analysis. All three patients who did not clear ctDNA had tumor relapse with a median time to relapse of 74 days (30-238), while 50% (7/14) of those who cleared ctDNA have remained disease free. Progression free survival was longer in patients who cleared ctDNA compared to those who did not (median 567 vs 74 d, p = 0.01).

**Conclusions:**

Although it is feasible to use ctDNA testing on a limited gene panel to identify patients with LA-NSCLC who are at high risk for disease recurrence following CRT, further studies will be necessary to optimize these assays before they can be used to inform clinical care in patients with lung cancer.

## Introduction

Lung cancer remains the leading cause of cancer deaths worldwide (1). Non-small cell lung cancer (NSCLC) accounts for 85% of lung cancer diagnoses (2). Approximately 27% of patients with NSCLC present with locally advanced disease (3), which is typically managed with concurrent chemoradiotherapy (CRT) (4). The PACIFIC trial showed improved overall survival in this patient population with the addition of consolidation durvalumab. However, a substantial proportion of patients with locally advanced NSCLC still recur with metastatic disease despite receiving CRT and consolidation durvalumab with curative intent (5). Additionally, a subset of patients may be cured by CRT alone without the need for consolidation durvalumab (6), which is associated with a 30.5% grade three or higher adverse event rate (5). Prognostic biomarkers therefore have the potential to identify patients who are likely to benefit from treatment intensification or de-escalation.

Tumor cells release circulating tumor DNA (ctDNA) into the bloodstream (7), and it is possible to identify mutations relevant for tumor development, progression, and resistance to therapy through ctDNA analyses. Additionally, the utility of ctDNA testing in informing prognosis in patients with early stage and locally advanced NSCLC (LA-NSCLC) has previously been described (8, 9). Since ctDNA testing has the potential to identify patients at high risk for recurrence following completion of concurrent CRT for LA-NSCLC, it may help identify patients that are likely to benefit more from escalation of treatment intensity (10). Past studies with ctDNA have, however, used large fixed hybrid gene capture panels that require prior knowledge of genomic alterations in a tumor (9, 10). Since this approach requires upfront sequencing of a tumor and construction of customized gene panels, its large-scale clinical application is limited. Here, we investigate the feasibility of using a commercial fixed gene panel based ctDNA testing approach, which does not require *a priori* knowledge of a tumor’s genomic profile, to predict outcomes in patients with locally advanced NSCLC. Based on results from previous studies, we hypothesized that outcomes in patients with locally advanced CRT are poorer when treatments fail to clear variants detected through pre-treatment ctDNA testing.

## Materials and Methods

Patients with histologically confirmed NSCLC with stage II or III NSCLC, who were not surgical candidates, and were eligible for curative intent treatment with concurrent chemotherapy and radiation were eligible for participation in this study. Patients were staged by the AJCC 8th edition TNM system. Patients receiving induction or consolidation chemotherapy and consolidation immune checkpoint inhibitors (ICIs) were also included. Progression following CRT was defined based on imaging, and when required, a biopsy at the discretion of the treating provider.

**Figure 1:**
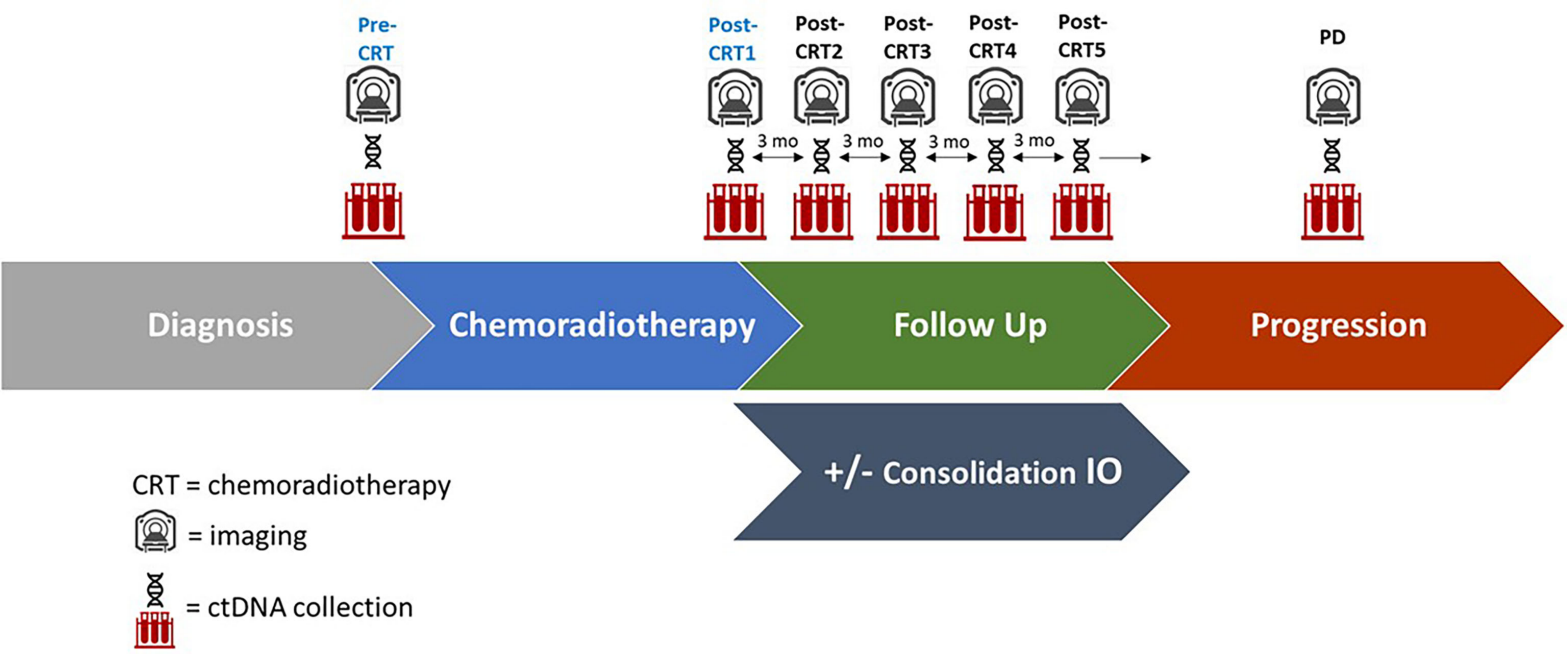
Study Schema of ctDNA collection, imaging, and follow up. Plasma for ctDNA was collected at the time of chemoradiotherapy initiation (pre-CRT), at completion of chemoradiotherapy (post-CRT1), and at quarterly follow up appointments for 12 months (post-CRT2,3,4, and 5 respectively), as well as at the time of disease progression (PD) if it occurred. A subset of patients received consolidation immune checkpoint inhibitors (IO). Imaging was performed every three months following completion of CRT.

Plasma for ctDNA testing was collected within 2 weeks of CRT initiation (pre-CRT), CRT completion (post-CRT1), quarterly follow up appointments for 12 months (post-CRT2, 3, 4, and 5 respectively) after CRT completion, and at the time of disease progression (PD) (**Figure 1** and **Supplementary Table**). All samples were collected with informed consent in accordance with the Declaration of Helsinki of 2013. Twenty mL of blood was collected in cell-free DNA blood collection (Streck) tubes. Tubes were labeled with specimen ID, subject ID, collection date and time point and shipped to Inivata (7020 Kit Creek Road, Suite 140, Research Triangle Park, NC 27560). ctDNA analysis was performed using the InVisionFirst^®^-Lung panel, to detect the presence of single nucleotide variations, insertions, deletions, and copy number alterations in 36 cancer-related genes, which are frequently altered in lung cancer (**Supplementary Figure 1**). Full assay details have been described previously (11–13). In brief, 36 cancer related genes were sequenced using gene-specific primers designed for hotspots and entire coding regions of interest. Next generation sequencing libraries were prepared from 2,000–16,000 amplifiable copies of the genome using a two-step PCR amplification process incorporating replicate and patient-specific barcodes and Illumina sequencing adaptors. Samples were pooled to generate a library of 12nM. 1.8 pM libraries were sequenced on the Illumina NextSeq 500, with sequencing files analyzed using Inivata’s proprietary Somatic Mutation Analysis (ISoMA) and FUSP pipelines.

Clearance of ctDNA was defined as the absence of non-germline pre-CRT variants at post-CRT1. Germline mutations were defined as a mutation allelic frequency (AF) greater than 40% detected at all time points (14, 15). Patients without detectable pre-CRT variants or no post-CRT1 samples were excluded from analysis. Maximum, mean, and median AF for all mutations detected in a sample were described, at each time point. All studies were undertaken in compliance with ethical principles established in the International Conference on Harmonization Good Clinical Practice and the Declaration of Helsinki. The study was approved by the Washington University in St. Louis Institutional Review Board. All statistical analyses were performed using GraphPad Prism (San Diego, CA; RRID : SCR_002798).

## Results

**Figure 2:**
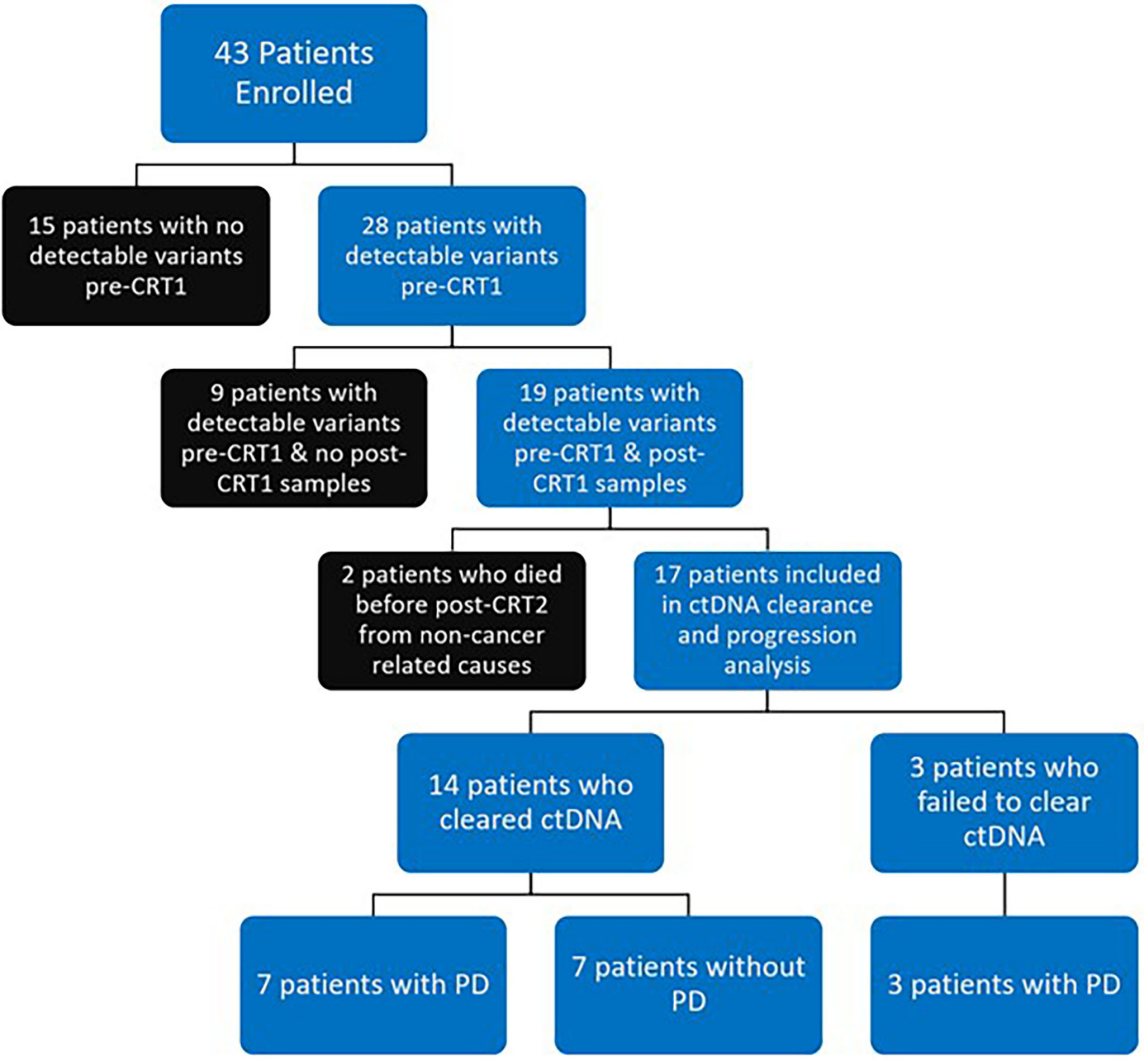
Flow Diagram of Patient Disposition: 43 patients were prospectively enrolled. 28 of 43 patients (65%) had detectable variants at time of chemoradiotherapy (CRT) initiation (pre-CRT). 19 of 43 patients (44%) had detectable variants at pre-CRT and had samples collected at completion of CRT (post-CRT1) and were included in final analysis. 2 of 19 patients (one who cleared ctDNA, one who did not) died from non-cancer related causes before the first visit post completion of CRT (post-CRT2) and were excluded from analysis on clearance. Of the 17 patients who were included in analysis of ctDNA clearance and disease progression (PD), 14 (82%) had clearance of ctDNA, with 7 having PD and 7 remaining free from progression. All 3 patients who did not clear ctDNA had PD.

A total of 43 patients were prospectively enrolled between September 2017 and October 2019. Ten patients were enrolled from Institut Gustave Roussy and 33 patients from Washington University School of Medicine in St. Louis. Twenty eight of 43 patients (65%) had detectable variants on pre-CRT ctDNA testing. Nineteen of 43 patients (44%) had detectable pre-CRT variants and post-CRT1 samples collected and were included in the final analysis (**Figure 2**). In this cohort of 19 patients (**Table 1**), the median age at diagnosis was 65 years (range 43 - 82). The majority of patients were smokers (16/19, 84%), male (12 of 19, 63%), and were diagnosed with stage IIIB disease (10 of 19, 53%). Nine patients (47%) were diagnosed with squamous cell carcinoma, seven (37%) with adenocarcinoma, and three (16%) with poorly differentiated or NSCLC not otherwise specified. All 19 patients received weekly carboplatin and paclitaxel chemotherapy. Ten of 19 (53%) received no consolidation ICIs, while 8 (42%) patients received an ICI (6 patients received durvalumab and 2 received atezolizumab).

**Table 1:** Baseline Characteristics.

Characteristic	Total Patients	Cleared ctDNA	Did not clear ctDNA
Number of Patients	19	14	3
Age at Diagnosis (years):	65 (43 - 82)	65 (43 - 78)	78 (62 - 82)
Gender (male):	12/19 (63%)	9/14 (64%)	2/3 (67%)
Tobacco Use:	16/19 (84%)	13/14 (93%)	2/3 (67%)
			
Stage:			
IIA	1/19 (5%)	0/14 (0%)	1/3 (33%)
IIIA	7/19 (37%)	5/14 (36%)	1/3 (33%)
IIIB	10/19 (52%)	8/14 (57%)	1/3 (33%)
IIIC	1/19 (5%)	1/14 (7%)	0/3 (0%)
Histology:			
Squamous	9/19 (47%)	6/14 (43%)	2/3 (67%)
Adenocarcinoma	7/19 (37%)	5/14 (36%)	1/3 (33%)
NSCLC NOS*	3/19 (16%)	3/14 (21%)	0/3 (0%)
			
Chemotherapy:			
Carboplatin + Paclitaxel	19/19 (100%)	14/14 (100%)	3/3 (100%)
Consolidation Immunotherapy:			
None	10/19 (53%)	7/14 (50%)	1/3 (33%)
Atezolizumab	2/19 (10%)	1/14 (7%)	1/3 (33%)
Durvalumab	6/19 (32%)	5/14 (36%)	1/3 (33%)
Unknown	1/19 (5%)	1/14 (7%)	0/3 (0%)

Nineteen patients had detectable pre-CRT variants and post-CRT1 samples collected and were included in the final analysis. Two of 19 patients died from non-cancer related causes before post-CRT2 and were excluded from further analysis on disease progression (1 cleared ctDNA, 1 did not).

*NSCLC NOS, non-small cell lung cancer not otherwise specified.

**Figure 3:**
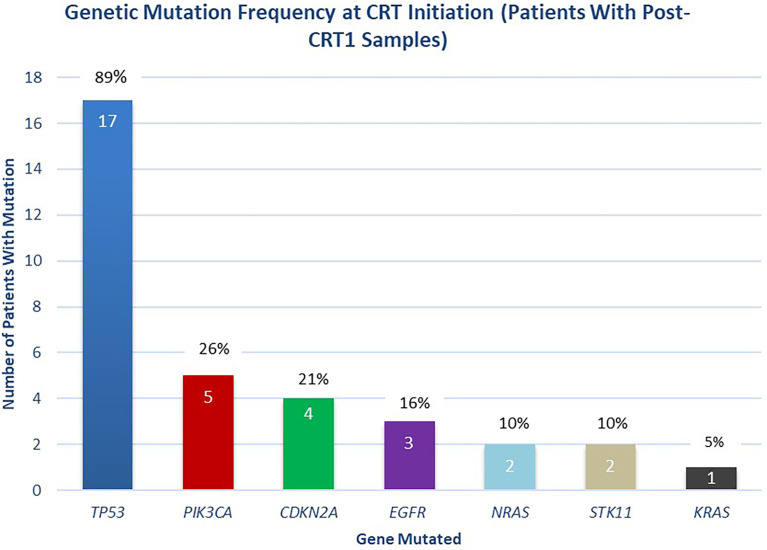
Distribution of Mutations Detected at Initiation of Chemoradiotherapy. Distribution of mutations detected at the initiation of chemoradiotherapy (pre-CRT), in patients with post-CRT1 samples collected (n = 19 patients).

A median of two mutations per sample (range 1-5) were detected with a median mean AF of 0.53% (range 0.05-16.28%) in pre-CRT samples. 100% (46 of 46) of mutations in the cohort of interest were non-synonymous. In pre-CRT samples from the 19 patients included in the final analysis, mutations in *TP53* were the most frequently detected (17/19, 89%), followed by mutations in *PIK3CA* (5/19, 26%), *CDKN2A* (4/19, 21%), and *EGFR* (3/19, 16%) (**Figure 3**).

**Figure 4:**
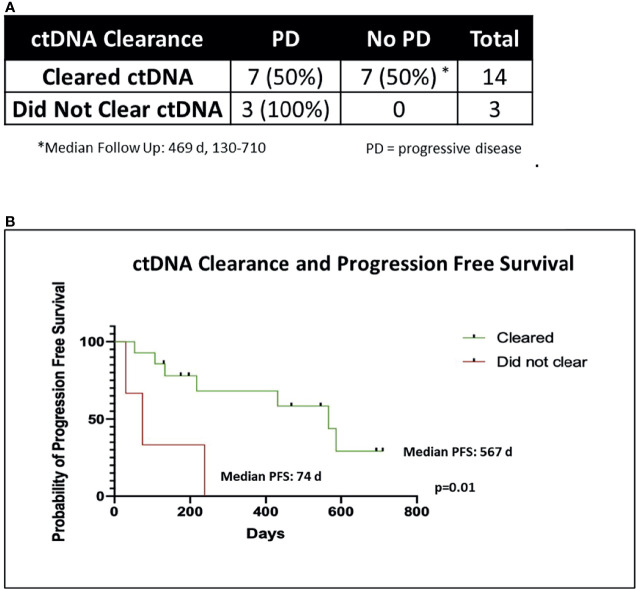
ctDNA clearance and freedom from progression. **(A)** Chi-Square table of ctDNA clearance and disease progression (PD). Two of 19 patients died of non-cancer related causes before post-CRT2 and were excluded from analysis of disease progression, leaving 17 patients. **(B)** Kaplan-Meier graph of progression free survival (PFS) in patients who cleared ctDNA (n = 14) versus those who did not (n = 3), using the Mantel Cox log rank test.

Two of 19 patients died from non-cancer related causes (myocardial infarction, acute respiratory distress syndrome) before post-CRT2 and were excluded from further analysis (1 cleared ctDNA, another did not). Three patients failed to clear ctDNA at post-CRT1 and 14 patients cleared ctDNA. All the 3 patients that failed to clear eventually progressed with a median time to progression of 74 days (range 30-238). In contrast, 7 of the 14 patients that cleared ctDNA at post-CRT1, remained disease free at the time of this analysis with a median follow-up of 469 days (range 130-710). The median time to progression in the seven patients who cleared ctDNA and later progressed was 217 days (range 53-587 days). In all patients who cleared ctDNA, the median PFS was 567 days, compared to a median PFS of 74 days in patients who did not clear (p = 0.01) (**Figure 4**). Of the 24 patients who did not have complete biomarker data, 1 died from non-cancer related causes before post-CRT2 and 2 patients were lost to follow up. Of the remaining 21 patients, 10 had disease progression (median time to progression 182 days, range 29 - 522) and 11 have not (median follow up 453 days, range 147 – 1099).

Of the 14 patients who cleared ctDNA, one patient’s consolidation therapy status was unknown, leaving 13 with known consolidation status in this group. Six of 13 patients who cleared ctDNA received consolidation ICIs, of which two had disease progression, with four remaining free from progression. In the seven of 13 patients who cleared ctDNA and did not receive consolidation ICIs, five had had disease progression, with two remaining progression free.

**Figure 5:**
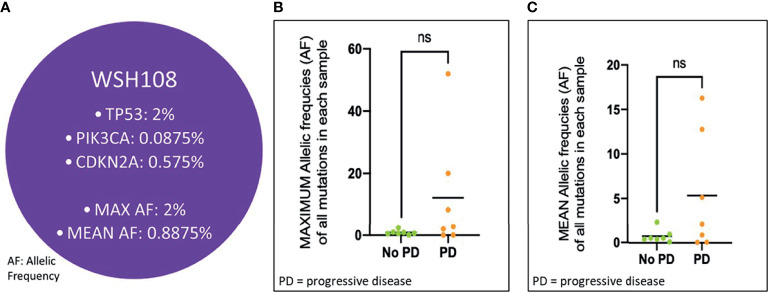
Mean and Max Allelic Frequency in Patients at CRT initiation who Cleared ctDNA, Stratified by Progression. **(A)** Schematic representation of a patient’s (WSH108) pre-CRT sample. Allelic frequency (AF) for each mutation detected in the sample was used for determining maximum and mean AF values. Each of these values across patients who progressed vs. did not progress were compared in the cohort of patients clearing ctDNA using a Student’s t-test, as depicted in **(B, C)** ns, not significant.

In the 14 patients who cleared ctDNA at post-CRT1, the pre-CRT median mean AF was 0.71% (range, 0.06 - 16.28%), with a median maximum AF of 0.90% (0.06 - 51.95%). In the seven of 14 patients that cleared ctDNA and later progressed, the median mean AF at pre-CRT was 2.11% (0.06 - 16.28%), with a median maximum AF of 2.80% (0.06 - 51.95%). In the seven of 14 patients that cleared and have not progressed, the median mean AF at pre-CRT was 0.52% (0.10 - 2.33%), and the median maximum AF was 0.63% (0.10 - 2.35%). There was no statistically significant difference in mean or maximum AF at pre-CRT in patients who progressed versus those who did not progress (p >0.05) (**Figure 5**). In the three patients who did not clear at post-CRT1, the pre-CRT median mean AF was 0.54% (0.26 - 0.72%) and the median maximum AF was 1.27% (0.47 - 1.61%). At post-CRT1, the median mean AF was 0.26% (0.11 - 1.89%) and the median maximum AF was 0.42% (0.17 - 5.55%).

New variants (non-pre-CRT1) were observed in eight (42%) of patients during follow-up with a median AF of 0.32% (range 0.03 - 15.14%). Of the eight patients with new mutations, six progressed and two did not. Of the seven of 14 patients who cleared ctDNA that later progressed, three had new mutations with a median AF of 0.07% (range 0.03 - 0.46%). In the three patients that did not clear ctDNA, all patients demonstrated new mutations at post-CRT1 or later, in addition to the pre-CRT mutations, with a median AF of 3.66% (range 0.17 - 15.14%) in the new variants.

## Discussion

To our knowledge, this is the first study utilizing commercially available ctDNA testing on a limited gene panel, instead of customized patient-specific gene panels, to show that detection of residual ctDNA following concurrent CRT in patients with locally advanced NSCLC is associated with a poor prognosis. All patients that failed to clear ctDNA following CRT in our analysis demonstrated disease progression. In addition, time to progression was much shorter in patients that failed to clear ctDNA, compared to patients that cleared ctDNA and eventually progressed (median 74 days vs 217 days). Furthermore, half the patients that cleared ctDNA following CRT continue to remain disease free at the time of this reporting.

Clearance of ctDNA following treatment, whether surgical or CRT, has been associated with improved prognosis in several other studies including lung cancer (10, 16–18). These studies however, used customized gene panels, the design of which required *a priori* tumor sequencing. In the study by Moding et al., which utilized personalized profiling by deep sequencing (CAPP-Seq) ctDNA analysis of 65 patients receiving CRT for locally advanced NSCLC, patients with undetectable ctDNA following CRT had improved outcomes relative to those with detectable ctDNA, regardless of whether they received consolidation ICI (10). Patients with detectable ctDNA following CRT who received consolidation ICIs had significantly improved outcomes than those who did not receive an ICI. The results from these studies, in addition to ours, support ctDNA clearance as a predictive biomarker in patients with locally advanced NSCLC. While previous studies have demonstrated the prognostic value of ctDNA clearance using customized gene panels, our study suggests that it could be feasible to utilize commercially available gene panels for the same purpose, but systematic testing through well-designed clinical trials are needed to further evaluate this.

The advantage of utilizing such commercial gene panel tests is that they are easy to incorporate into clinical practice and are not associated with the challenges and high costs associated with upfront tumor sequencing and design of customized gene panels. However, the successful translation of assays that utilize ctDNA sequencing on a limited gene panel to the clinic will require addressing certain crucial limitations that we identified in our analysis. For instance, the limited sequencing space and number of genes included on limited panels can potentially misclassify patients not clearing ctDNA as having cleared ctDNA. In this regard, the 65% of patients with mutations detected post-CRT in our study could be erroneously low due to the decreased sensitivity of our limited gene sequencing platform. Additionally, as is the limitation with all ctDNA assays that lack sequencing data from tumor tissue or peripheral blood mononuclear cells, it is difficult to distinguish tumor derived ctDNA from DNA originating from clonal hematopoiesis of indeterminate potential (CHIP).

Nevertheless, despite these limitations, the most commonly detected mutations in our study were in genes that are frequently mutated in lung cancer, which supports the conclusions reported. Mutations in *TP53*, *PIK3CA*, *CDKN2A*, *KRAS*, and *EGFR* were the most commonly detected, which is similar to known frequencies of mutations in lung cancer from tumor biopsies (19–22). Eight of the 17 patients (47%) had new emerging variants, defined as not present in the pre-CRT samples, with six of these patients (75%) developing relapse. It is possible that some of these variants were present in the pre-CRT and post-CRT1 samples, but at a level of detection that is lower than the limit of detection for the assay, leading to the possible misclassification of these patients into the “ctDNA cleared” category. We anticipate that advances in ctDNA sequencing technology in the near future may help address these limitations of liquid biopsies and improve the performance of this assay. Since it is also possible that such emerging variants could have been acquired by a tumor during the course of treatment, assays with fixed panels comprising of several frequently mutated genes in lung cancer may have an inherent advantage over assays using customized panels guided by tissue sequencing for identifying patients with emerging variants early in the course of their treatment, which may potentially be used for treatment changes.

The findings of our study are best viewed as exploratory given the limited sample size. Apart from the small sample size, the lack of longitudinal post-CRT variant data at all time points for all participants, and the fact that only a limited number of patients received consolidation immunotherapy in our study, which is currently the standard of care - limited our ability to investigate whether ctDNA clearance has the ability to identify patients most likely to benefit from immunotherapy.

In summary, results from this study demonstrate that it is feasible to employ ctDNA testing utilizing an “off the shelf” gene panel assay to identify LA-NSCLC patients who are at high risk for disease recurrence following CRT. Our results suggest that the failure to clear ctDNA after CRT is a poor prognostic factor for early progression. However, this approach is not without certain limitations that can potentially be circumvented by advances in sequencing technology, utilizing matched peripheral blood cell sequencing, and using expanded gene panels. Whether such assays can be used to inform clinical practice merits future investigation. Prospective validation of our results in larger studies is necessary before these findings can be translated to the clinic.

## Data Availability Statement

The original contributions presented in the study are included in the article/supplementary material, further inquiries can be directed to the corresponding author/s.

## Ethics Statement

The studies involving human participants were reviewed and approved by Washington University in St. Louis Institutional Review Board. The patients/participants provided their written informed consent to participate in this study.

## Author Contributions

BK: Formal Analysis, Investigation, Data Curation, Writing – Original Draft, Writing – Review & Editing, Visualization. LM: Conceptualization, Methodology, Validation, Formal Analysis, Investigation, Resources, Data Curation, Writing – Review & Editing, Project Administration, Funding Acquisition. SD: Methodology, Validation, Formal analysis, Investigation, Data Curation, Writing – Original Draft, Writing – Review and Editing, Visualization, Project Administration. MA: Formal Analysis, Investigation, Data Curation, Writing – Review & Editing. SW: Methodology, Investigation, Writing – Review & Editing. KP: Formal Analysis, Investigation, Data Curation, Writing – Review & Editing. JW: Methodology, Investigation, Writing – Review & Editing. AB: Methodology, Investigation, Writing – Review & Editing. KH: Methodology, Writing – Review & Editing, Project Administration, Funding Acquisition. CK: Methodology, Writing – Review & Editing, Funding Acquisition. CM: Methodology, Writing – Review & Editing, Project Administration, Funding Acquisition. RG: Methodology, Validation, Investigation, Resources, Writing – Review & Editing. BB: Conceptualization, Methodology, Validation, Investigation, Data Curation, Writing – Review & Editing, Supervision, Project Administration. DM: Conceptualization, Methodology, Validation, Investigation, Resources, Data Curation, Writing – Original Draft, Writing – Review & Editing, Visualization, Supervision, Project Administration, Funding Acquisition. All authors contributed to the article and approved the submitted version.

## Conflict of Interest

LM: Lectures and educational activities: Bristol-Myers Squibb, Tecnofarma, AstraZeneca, Roche, Takeda. Consulting/advisory role: Roche, Takeda. Research Grants: Bristol-Myers Squibb, Boehringer Ingelheim. Travel, Accommodations, Expenses: Bristol-Myers Squibb, Roche. Others: International Mentorship Program funded by AstraZeneca. SW: Research grant support from -2% effort on “Duke-UNC Wash U Partnership for Early Phase Clinical Trials in Cancer” 1UM1 CA186704-01 grant as mentored faculty, DSMB Chair for Hoosier Cancer Research Network study, and institutional PI for studies with support from Hoffmann-La Roche Ltd, Ariad, Pfizer Pharmaceuticals, Inc., Hengrui Therapeutics, Xcovery, EMD Serono Research & Development Institute, Inc., Checkpoint Therapeutics, Inc., Genentech, Inc., Lilly, Stemcentrx, Inc., Ignyta, Inc., Bristol-Myers Squibb Pharmaceutical, Synermore Biologics Co., Ltd., Novartis Pharmaceuticals Corporation, Merck & Company, Inc., NewLink Genetics Corporation, and Celgene.

JW: Advisory board for Novocure, Consultant for Novoure, Employment for Millipore (Spouse), Travel/Expenses from Halozyme. KH: employee and stockholder for Inivata. CK: employee and stockholder for Inivata. CM: employee and stockholder for Inivata.

RG: Advisory Board for Achilles, Consulting for GenePlus, Horizon Pharmaceuticals (Spouse). BB: Research support from 4D Pharma, Abbvie, Amgen, Aptitude Health, AstraZeneca, BeiGene, Blueprint Medicines, BMS, Boehringer Ingelheim, Celgene, Cergentis, Cristal Therapeutics, Daiichi-Sankyo, Eli Lilly, GSK, Inivata, Janssen, Onxeo, OSE immunotherapeutics, Pfizer, Roche-Genentech, Sanofi, Takeda, Tolero Pharmaceuticals. DM: Advisory board for Abbvie, Takeda, PharmaMar, Gilead, Boehringer Ingelheim; Consultant for Abbvie, Takeda, Boehringer Ingelheim, PharmaMar, Gilead.

The study received funding from Inivata. The funder had the following involvement in the study: study design, analysis and data interpretation, the writing of this article, and the decision to submit it for publication.

## Publisher’s Note

All claims expressed in this article are solely those of the authors and do not necessarily represent those of their affiliated organizations, or those of the publisher, the editors and the reviewers. Any product that may be evaluated in this article, or claim that may be made by its manufacturer, is not guaranteed or endorsed by the publisher.
