# Supplementary material for: Exploring the Feasibility of Utilizing Limited Gene Panel Circulating Tumor DNA Clearance as a Biomarker in Patients With Locally Advanced Non-Small Cell Lung Cancer
Source: Front Oncol. 2022 Mar 28;12:856132. doi: 10.3389/fonc.2022.856132 (PMC9000093; doi:10.3389/fonc.2022.856132)

**SUPPLEMENTARY FIGURES**

**1. InvisionFirst-Lung Full Gene Panel**

The 36 gene panel includes single nucleotide variants, inframe deletions and copy number variants in cancer-related genes.


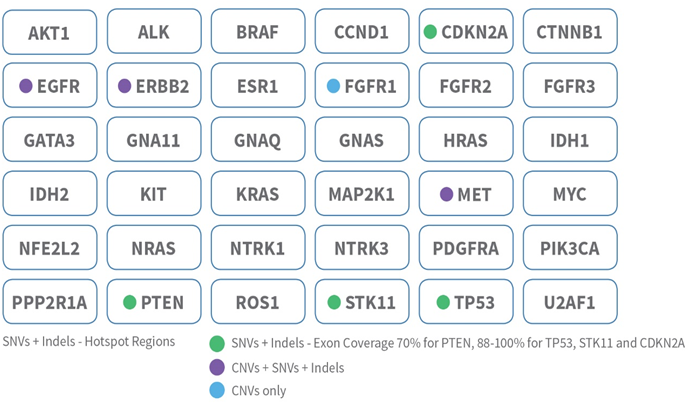

Supplement: Supplementary file 1 [file DataSheet_1.docx]
