# Supplementary material for: Exploring the Feasibility of Utilizing Limited Gene Panel Circulating Tumor DNA Clearance as a Biomarker in Patients With Locally Advanced Non-Small Cell Lung Cancer
Source: Front Oncol. 2022 Mar 28;12:856132. doi: 10.3389/fonc.2022.856132 (PMC9000093; doi:10.3389/fonc.2022.856132)
Supplement: Supplementary file 2 [file Table_1.docx]

**Supplementary Table 1**

Here we report the raw genomic data collected for each patient included in the study (both those included in the final analysis and those missing data). The third column, “QC Flag” reports whether or not the genomic data passed quality control on initial data check. The last tab, “Clearance”, reports whether the patient cleared ctDNA (yes), did not clear (no), or did not have complete data and was not included in the final analysis (NA).

| **Patient ID** | **Timepoint** | **QC Flag** | **Mutations Detected** | **Code** | **Gene** | **Mutation** | **Frequency Percentage** | **Clearance** |
| --- | --- | --- | --- | --- | --- | --- | --- | --- |
| WSH100 | pre-CRT | PASS | Yes | mis_chr17_7673802_C_T | TP53 | p.R273H | 0.46552 | No |
|  |  |  |  | mis_chr7_55191822_T_G | EGFR | p.L858R | 0.06034 | No |
|  | post-CRT1 | PASS | Yes | mis_chr17_7673802_C_T | TP53 | p.R273H | 0.42135 | No |
|  |  |  |  | mis_chr7_55191822_T_G | EGFR | p.L858R | 0.09831 | No |
|  | PD | PASS | Yes | mis_chr17_39711955_C_T | ERBB2 | p.S310F | 3.35681 | No |
|  |  |  |  | mis_chr17_7673776_G_A | TP53 | p.R282W | 3.96714 | No |
|  |  |  |  | mis_chr17_7673802_C_T | TP53 | p.R273H | 0.58685 | No |
|  |  |  |  | mis_chr7_55191822_T_G | EGFR | p.L858R | 2.52347 | No |
| WSH102 | pre-CRT | PASS | No | No call for this sample | NA | NA | NA | NA |
|  | post-CRT1 | PASS | No | No call for this sample | NA | NA | NA | NA |
|  | post-CRT1 | PASS | No | No call for this sample | NA | NA | NA | NA |
|  | PD | PASS | No | No call for this sample | NA | NA | NA | NA |
| WSH103 | pre-CRT | PASS | Yes | mis_chr12_25245351_C_A | KRAS | p.G12C | 0.2125 | NA |
|  |  |  |  | mis_chr17_7674264_G_C | TP53 | p.H233Q | 0.225 | NA |
|  | post-CRT1 | FAIL | Yes | mis_chr12_25245351_C_A | KRAS | p.G12C | 0.19167 | NA |
| WSH105 | pre-CRT | PASS | Yes | del_chr17_7676031_AAG_- | TP53 | p.112-113:GF/G | 1.6125 | No |
|  |  |  |  | mis_chr17_7674268_A_T | TP53 | p.I232N | 0.25 | No |
|  |  |  |  | mis_chr17_7675086_A_T | TP53 | p.C176S | 0.1625 | No |
|  |  |  |  | mis_chr3_179218303_G_A | PIK3CA | p.E545K | 0.1375 | No |
|  | post-CRT1 | PASS | Yes | del_chr17_7674190_T_- | TP53 | p.258:E/X | 0.41791 | No |
|  |  |  |  | del_chr17_7676031_AAG_- | TP53 | p.112-113:GF/G | 5.55224 | No |
|  |  |  |  | mis_chr17_7674268_A_T | TP53 | p.I232N | 1.38806 | No |
|  |  |  |  | mis_chr17_7675086_A_T | TP53 | p.C176S | 0.20896 | No |
| WSH106 | pre-CRT | PASS | Yes | mis_chr3_179218303_G_A | PIK3CA | p.E545K | 0.06818 | yes |
|  | post-CRT1 | PASS | No | No call for this sample | NA | NA | NA | yes |
|  | post-CRT2 | PASS | No | No call for this sample | NA | NA | NA | yes |
|  | post-CRT4 | PASS | No | No call for this sample | NA | NA | NA | yes |
|  | post-CRT5 | PASS | No | No call for this sample | NA | NA | NA | yes |
|  | PD | PASS | Yes | mis_chr3_179218303_G_A | PIK3CA | p.E545K | 0.15625 | yes |
|  | PD | PASS | No | No call for this sample | NA | NA | NA | yes |
| WSH107 | pre-CRT | PASS | Yes | mis_chr12_25245351_C_A | KRAS | p.G12C | 22.11875 | NA |
|  |  |  |  | mis_chr17_7673806_C_A | TP53 | p.V272L | 4.4 | NA |
| WSH108 | pre-CRT | PASS | Yes | mis_chr17_7673767_C_T | TP53 | p.E285K | 2 | yes |
|  |  |  |  | mis_chr3_179218303_G_A | PIK3CA | p.E545K | 0.0875 | yes |
|  |  |  |  | mis_chr9_21971117_G_A | CDKN2A | p.P81L | 0.575 | yes |
|  | post-CRT1 | PASS | No | No call for this sample | NA | NA | NA | yes |
|  | post-CRT2 | PASS | No | No call for this sample | NA | NA | NA | yes |
|  | post-CRT3 | PASS | Yes | mis_chr19_1220702_C_A | STK11 | p.S240* | 0.45643 | yes |
| WSH109 | pre-CRT | PASS | Yes | mis_chr17_7675993_C_A | TP53 | NA | 0.45894 | yes |
|  |  |  |  | mis_chr19_1221212_G_T | STK11 | NA | 0.36232 | yes |
|  | post-CRT1 | PASS | No | No call for this sample | NA | NA | NA | yes |
|  | post-CRT1 | PASS | Yes | mis_chr17_7674238_C_T | TP53 | p.C242Y | 0.30374 | yes |
|  | post-CRT3 | PASS | Yes | mis_chr17_7674238_C_T | TP53 | p.C242Y | 0.5 | yes |
|  | post-CRT4 | PASS | Yes | mis_chr17_7674238_C_T | TP53 | p.C242Y | 0.31818 | yes |
|  | post-CRT3 | PASS | No | No call for this sample | NA | NA | NA | yes |
| WSH111 | pre-CRT | PASS | Yes | mis_chr17_7673793_G_C | TP53 | p.A276G | 0.78704 | No |
|  |  |  |  | mis_chr17_7675185_C_T | TP53 | p.V143M | 0.09259 | No |
|  |  |  |  | mis_chr1_114713908_T_C | NRAS | p.Q61R | 1.27315 | No |
|  | post-CRT1 | PASS | Yes | mis_chr17_7673793_G_T | TP53 | p.A276D | 0.16667 | No |
|  |  |  |  | mis_chr17_7675185_C_T | TP53 | p.V143M | 0.05556 | No |
|  | post-CRT2 | PASS | Yes | mis_chr17_7673793_G_C | TP53 | p.A276G | 20.18519 | No |
|  |  |  |  | mis_chr1_114713908_T_C | NRAS | p.Q61R | 25.36111 | No |
|  |  |  |  | mis_chr9_21974680_G_A | CDKN2A | p.Q50* | 7.98148 | No |
|  | post-CRT3 | PASS | Yes | mis_chr17_7673793_G_C | TP53 | p.A276G | 24.56897 | No |
|  |  |  |  | mis_chr17_7675185_C_T | TP53 | p.V143M | 0.14368 | No |
|  |  |  |  | mis_chr1_114713908_T_C | NRAS | p.Q61R | 31.56609 | No |
|  |  |  |  | mis_chr9_21974680_G_A | CDKN2A | p.Q50* | 15.14368 | No |
|  | post-CRT2 | FAIL | No | No call for this sample | NA | NA | NA | No |
| WSH112 | pre-CRT | PASS | Yes | amplification_FGFR1 | FGFR1 | NA | NA | yes |
|  |  |  |  | del_chr17_7675084_G_- | TP53 | p.176:C/X | 8.33333 | yes |
|  |  |  |  | del_chr9_21971018_GG_- | CDKN2A | p.114:P/X | 4.87179 | yes |
|  |  |  |  | mis_chr17_7675190_C_T | TP53 | p.C141Y | 7.08791 | yes |
|  |  |  |  | mis_chr8_127738292_G_C | MYC | p.R25S | 0.20147 | yes |
|  | post-CRT1 | PASS | No | No call for this sample | NA | NA | NA | yes |
| WSH113 | pre-CRT | PASS | Yes | del_chr17_7674206_TGAGGATGG_- | TP53 | p.250-253:PILT/P | 0.0551 | yes |
|  | post-CRT1 | PASS | No | No call for this sample | NA | NA | NA | yes |
|  | post-CRT1 | PASS | No | No call for this sample | NA | NA | NA | yes |
|  | post-CRT2 | PASS | No | No call for this sample | NA | NA | NA | yes |
|  | post-CRT3 | PASS | Yes | del_chr17_7675994_CGTGCAAG_- | TP53 | p.123-125:TCT/X | 0.03289 | yes |
|  | post-CRT4 | PASS | Yes | del_chr17_7675994_CGTGCAAG_- | TP53 | p.123-125:TCT/X | 0.02959 | yes |
|  | post-CRT5 | PASS | No | No call for this sample | NA | NA | NA | yes |
| WSH114 | pre-CRT | PASS | Yes | ins_chr19_1207119_-_A | STK11 | p.69:S/SX | 0.85 | yes |
|  |  |  |  | ins_chr19_1207121_-_G | STK11 | p.70:E/GX | 0.1375 | yes |
|  |  |  |  | mis_chr17_7674217_C_A | TP53 | p.R249M | 0.3125 | yes |
|  |  |  |  | mis_chr19_1221307_G_A | STK11 | p.D277N | 0.8125 | yes |
|  | post-CRT1 | PASS | No | No call for this sample | NA | NA | NA | yes |
|  | post-CRT3 | PASS | Yes | mis_chr19_1221307_G_A | STK11 | p.D277N | 0.53571 | yes |
|  | post-CRT4 | PASS | No | No call for this sample | NA | NA | NA | yes |
|  | post-CRT5 | PASS | Yes | mis_chr19_1221307_G_A | STK11 | p.D277N | 0.42373 | yes |
| WSH115 | pre-CRT | PASS | Yes | mis_chr17_7673796_C_A | TP53 | p.C275F | 2.8 | yes |
|  |  |  |  | mis_chr9_21971037_C_A | CDKN2A | p.D108Y | 1.4125 | yes |
|  | post-CRT1 | PASS | No | No call for this sample | NA | NA | NA | yes |
|  | post-CRT2 | PASS | No | No call for this sample | NA | NA | NA | yes |
|  | PD | PASS | Yes | mis_chr17_7673796_C_A | TP53 | p.C275F | 0.70359 | yes |
|  |  |  |  | mis_chr9_21971037_C_A | CDKN2A | p.D108Y | 0.34431 | yes |
| WSH116 | pre-CRT | FAIL | No | No call for this sample | NA | NA | NA | NA |
|  | post-CRT1 | FAIL | Yes | mis_chr17_7674947_A_G | TP53 | p.I195T | 0.68376 | NA |
|  | post-CRT1 | PASS | Yes | mis_chr17_7674947_A_G | TP53 | p.I195T | 0.41985 | NA |
|  | post-CRT1 | PASS | No | No call for this sample | NA | NA | NA | NA |
|  | post-CRT2 | PASS | Yes | mis_chr17_7674947_A_G | TP53 | p.I195T | 0.6338 | NA |
|  |  |  |  | mis_chr17_7675139_C_T | TP53 | p.R158H | 0.35211 | NA |
|  | post-CRT4 | PASS | Yes | mis_chr17_7674947_A_G | TP53 | p.I195T | 0.44118 | NA |
|  | post-CRT5 | PASS | Yes | mis_chr17_7674947_A_G | TP53 | p.I195T | 0.60811 | NA |
| WSH117 | pre-CRT | PASS | No | No call for this sample | NA | NA | NA | NA |
|  | post-CRT1 | PASS | Yes | del_chr7_55174773_GAATTAAGAGAAGCA_- | EGFR | p.746-750:ELREA/- | 0.01157 | NA |
|  |  |  |  | mis_chr17_7673788_G_T | TP53 | p.P278T | 0.02315 | NA |
|  | PD | PASS | No | No call for this sample | NA | NA | NA | NA |
| WSH118 | pre-CRT | PASS | No | No call for this sample | NA | NA | NA | NA |
|  | pre-CRT | PASS | No | No call for this sample | NA | NA | NA | NA |
|  | post-CRT1 | PASS | Yes | mis_chr17_7675185_C_T | TP53 | p.V143M | 0.21127 | NA |
| WSH119 | pre-CRT | PASS | No | No call for this sample | NA | NA | NA | NA |
|  | post-CRT1 | PASS | Yes | del_chr7_55174773_GAATTAAGAGAAGCA_- | EGFR | p.746-750:ELREA/- | 0.03788 | NA |
|  | post-CRT4 | PASS | Yes | del_chr7_55174773_GAATTAAGAGAAGCA_- | EGFR | p.746-750:ELREA/- | 0.0875 | NA |
|  | post-CRT5 | PASS | Yes | del_chr7_55174773_GAATTAAGAGAAGCA_- | EGFR | p.746-750:ELREA/- | 0.2439 | NA |
|  |  |  |  | mis_chr17_7676096_C_T | TP53 | p.W91* | 0.41463 | NA |
| WSH120 | pre-CRT | PASS | Yes | mis_chr1_114713908_T_A | NRAS | p.Q61L | 0.33019 | NA |
|  | post-CRT2 | PASS | No | No call for this sample | NA | NA | NA | NA |
|  | post-CRT3 | PASS | No | No call for this sample | NA | NA | NA | NA |
|  | post-CRT4 | PASS | No | No call for this sample | NA | NA | NA | NA |
| WSH121 | pre-CRT | PASS | Yes | mis_chr17_7675143_C_A | TP53 | p.V157F | 2.35 | yes |
|  |  |  |  | mis_chr19_1220372_G_T | STK11 | NA | 2.3125 | yes |
|  | post-CRT1 | PASS | No | No call for this sample | NA | NA | NA | yes |
|  | post-CRT4 | PASS | No | No call for this sample | NA | NA | NA | yes |
|  | post-CRT5 | PASS | No | No call for this sample | NA | NA | NA | yes |
| WSH122 | pre-CRT | PASS | No | No call for this sample | NA | NA | NA | NA |
|  | post-CRT1 | PASS | No | No call for this sample | NA | NA | NA | NA |
|  | post-CRT2 | PASS | Yes | mis_chr17_7673533_A_C | TP53 | NA | 7.2625 | NA |
|  | post-CRT3 | PASS | Yes | mis_chr17_7673533_A_C | TP53 | NA | 8.05687 | NA |
|  | post-CRT4 | PASS | No | No call for this sample | NA | NA | NA | NA |
| WSH123 | pre-CRT | PASS | Yes | mis_chr3_179199088_G_A | PIK3CA | p.R88Q | 0.14375 | NA |
|  | post-CRT1 | PASS | No | No call for this sample | NA | NA | NA | NA |
|  | post-CRT1 | PASS | No | No call for this sample | NA | NA | NA | NA |
| WSH124 | pre-CRT | PASS | No | No call for this sample | NA | NA | NA | NA |
|  | post-CRT1 | PASS | No | No call for this sample | NA | NA | NA | NA |
|  | post-CRT2 | PASS | No | No call for this sample | NA | NA | NA | NA |
|  | post-CRT3 | PASS | No | No call for this sample | NA | NA | NA | NA |
| WSH125 | pre-CRT | PASS | Yes | mis_chr17_7675185_C_T | TP53 | p.V143M | 0.20725 | NA |
| WSH126 | pre-CRT | PASS | No | No call for this sample | NA | NA | NA | NA |
|  | post-CRT1 | PASS | Yes | mis_chr19_1221294_C_T | STK11 | p.Y272Y | 0.46348 | NA |
|  | post-CRT3 | PASS | Yes | mis_chr17_7675085_C_T | TP53 | p.C176Y | 0.78067 | NA |
| WSH128 | pre-CRT | PASS | No | No call for this sample | NA | NA | NA | NA |
|  | post-CRT1 | PASS | Yes | del_chr15_66435093_CCTTGAGGCCT_- | MAP2K1 | p.49-53:RLEAF/RX | 0.07114 | NA |
|  |  |  |  | mis_chr17_7673802_C_A | TP53 | p.R273L | 0.20325 | NA |
|  | post-CRT2 | PASS | Yes | mis_chr17_7673802_C_A | TP53 | p.R273L | 0.225 | NA |
|  | post-CRT3 | PASS | Yes | mis_chr17_7673802_C_A | TP53 | p.R273L | 0.225 | NA |
|  |  |  |  | mis_chr17_7675076_T_C | TP53 | p.H179R | 0.4125 | NA |
| WSH129 | pre-CRT | PASS | No | No call for this sample | NA | NA | NA | NA |
| WSH130 | pre-CRT | PASS | Yes | del_chr9_21974803_TGCT_- | CDKN2A | p.8-9:SM/X | 2.3625 | yes |
|  |  |  |  | mis_chr10_87965321_C_A | PTEN | p.P354Q | 51.95 | yes |
|  |  |  |  | mis_chr17_7673787_G_T | TP53 | p.P278H | 3.38125 | yes |
|  |  |  |  | mis_chr7_55191822_T_G | EGFR | p.L858R | 3.71875 | yes |
|  |  |  |  | mis_chr9_21974820_G_C | CDKN2A | p.P3R | 2.3375 | yes |
|  | post-CRT1 | PASS | Yes | mis_chr10_87965321_C_A | PTEN | p.P354Q | 48.26633 | yes |
|  | PD | PASS | Yes | del_chr9_21974803_TGCT_- | CDKN2A | p.8-9:SM/X | 0.54913 | yes |
|  |  |  |  | mis_chr10_87965321_C_A | PTEN | p.P354Q | 51.48844 | yes |
|  |  |  |  | mis_chr17_7673787_G_T | TP53 | p.P278H | 0.75867 | yes |
|  |  |  |  | mis_chr7_55191822_T_G | EGFR | p.L858R | 0.78035 | yes |
|  |  |  |  | mis_chr9_21974820_G_C | CDKN2A | p.P3R | 0.53468 | yes |
|  | post-CRT2 | PASS | Yes | del_chr9_21974803_TGCT_- | CDKN2A | p.8-9:SM/X | 1.125 | yes |
|  |  |  |  | mis_chr10_87965321_C_A | PTEN | p.P354Q | 51.0375 | yes |
|  |  |  |  | mis_chr17_7673787_G_T | TP53 | p.P278H | 1.7625 | yes |
|  |  |  |  | mis_chr7_55191822_T_G | EGFR | p.L858R | 1.96875 | yes |
|  |  |  |  | mis_chr9_21974820_G_C | CDKN2A | p.P3R | 1.1375 | yes |
| WSH131 | pre-CRT | PASS | Yes | del_chr17_7674860_T_- | TP53 | p.224:E/X | 0.52402 | yes |
|  | post-CRT1 | PASS | No | No call for this sample | NA | NA | NA | yes |
|  | post-CRT2 | PASS | No | No call for this sample | NA | NA | NA | yes |
| WSH132 | pre-CRT | PASS | Yes | mis_chr17_7673787_G_C | TP53 | p.P278R | 8.83033 | yes |
|  |  |  |  | mis_chr3_179218303_G_A | PIK3CA | p.E545K | 20.01928 | yes |
|  |  |  |  | mis_chr3_179218315_G_C | PIK3CA | p.D549H | 19.99357 | yes |
|  | post-CRT1 | PASS | Yes | del_chr17_7675204_TTGGCAAAACATCTTGTTGAG_- | TP53 | p.130-136:LNKMFCQ/- | 0.07018 | yes |
|  | post-CRT1 | PASS | Yes | mis_chr17_7674908_T_A | TP53 | p.D208V | 0.20101 | yes |
|  | post-CRT2 | PASS | Yes | mis_chr3_179218303_G_A | PIK3CA | p.E545K | 0.12238 | yes |
| WSH133 | pre-CRT | PASS | No | No call for this sample | NA | NA | NA | NA |
|  | post-CRT1 | PASS | Yes | mis_chr12_25245350_C_G | KRAS | p.G12A | 0.05319 | NA |
| WSH135 | pre-CRT | PASS | Yes | mis_chr17_7673781_C_A | TP53 | p.R280I | 0.43554 | yes |
|  |  |  |  | mis_chr3_41224610_C_G | CTNNB1 | p.S33C | 0.0784 | yes |
|  |  |  |  | mis_chr7_55174015_G_C | EGFR | p.G719A | 0.4878 | yes |
|  |  |  |  | mis_chr7_55181312_G_T | EGFR | p.S768I | 0.62718 | yes |
|  | post-CRT1 | PASS | No | No call for this sample | NA | NA | NA | yes |
| WSH136 | pre-CRT | PASS | No | No call for this sample | NA | NA | NA | NA |
| WSH137 | pre-CRT | PASS | Yes | del_chr17_7674271_G_- | TP53 | p.231:T/X | 0.95238 | yes |
|  | post-CRT1 | PASS | Yes | mis_chr1_114713907_T_G | NRAS | p.Q61H | 0.1264 | yes |
| 1205 | post-CRT3 | PASS | Yes | TP53 mut p.392-393:SD/X (0,15%) | TP53 | p.392-393:SD/X | 0.15 | NA |
|  |  |  |  | TP53 mut p.294-299:EPHHEL/DX (0,22%) | TP53 | p.294-299:EPHHEL/DX | 0.22 | NA |
| 1346 | pre-CRT | FAIL | No | failed -low read depth, risk of false negatives- | NA | NA | NA | NA |
|  |  | PASS | Yes | CDKN2A mut p.105-109:DVRDA/X (0,08%) | CDNK2A | p.105-109:DVRDA/X | 0.08 | NA |
|  | PD | FAIL | No | failed, None detected | NA | NA | NA | NA |
| 1350 | pre-CRT | PASS | Yes | STK11 mut (I360T,1,09%) | STK11 | I360T | 1.09 | NA |
|  |  |  |  | TP53 mut (splice acceptor v 3,61%) | TP53 | splice acceptor v 3 | 3.61 | NA |
|  |  |  |  | FGFR2 mut (K660E, 0,66%) | FGFR2 | K660E | 0.66 | NA |
|  |  |  |  | NFE2L2 (L30F 0,55%) | NFE2L2 | L30F | 0.55 | NA |
|  | PD | PASS | Yes | TP53 mut (splice acceptor v 0,76%) | TP53 | splice acceptor v 0 | 0.76 | NA |
|  |  |  |  | FGFR2 mut (K660E, 0,6%) | FGFR2 | K660E | 0.6 | NA |
|  |  |  |  | PTEN mut (N31K, 0,71%) | PTEN | N31K | 0.71 | NA |
| 1378 | pre-CRT | PASS | Yes | TP53 mut (P278L, 0,10%) | TP53 | P278L | 0.1 | yes |
|  | NA | PASS | No | None detected | NA | NA | NA | yes |
|  | post-CRT1 | PASS | No | None detected | NA | NA | NA | yes |
|  | post-CRT2 | PASS | No | None detected | NA | NA | NA | yes |
|  | post-CRT3 | PASS | No | None detected | NA | NA | NA | yes |
|  | post-CRT4 | PASS | No | None detected | NA | NA | NA | yes |
| 1491 | NA | FAIL | No | Missing sample in inivata list? | NA | NA | NA | NA |
|  | post-CRT1 | FAIL | No | failed -low read depth, risk of false negatives- | NA | NA | NA | NA |
|  | post-CRT2 | PASS | Yes | TP53 mut (G266R, 0,28%) | TP53 | G266R | 0.28 | NA |
|  | post-CRT3 | PASS | No | None detected | NA | NA | NA | NA |
|  | post-CRT4 | PASS | No | None detected | NA | NA | NA | NA |
|  |  |  |  | None detected | NA | NA | NA | NA |
| 1499 | POST-CRT5 | PASS | Yes | TP53 mut (C176G, 0,73%) | TP53 | C176G | 0.73 | NA |
| 1586 | pre-CRT | PASS | Yes | KRAS mut (G12A, 1,99%) | KRAS | G12A | 1.99 | NA |
|  |  |  |  | TP53 mut (S241T, 0,47%) | TP53 | S241T | 0.47 | NA |
|  | PD | FAIL | No | Missing sample in inivata list? | NA | NA | NA | NA |
| 1675 | pre-CRT | PASS | Yes | KRAS mut (G12V, 1,9%) | KRAS | G12V | 1.9 | NA |
|  |  |  |  | TP53 mut (R158L, 0,68%) | TP53 | R158L | 0.68 | NA |
|  | NA | PASS | No | None detected | NA | NA | NA | NA |
|  |  |  |  | None detected | NA | NA | NA | NA |
|  | post-CRT1 | FAIL | No | Missing sample in inivata list? | NA | NA | NA | NA |
|  | PD | PASS | Yes | KRAS mut (G12V, 3,42%) | KRAS | G12V | 3.42 | NA |
|  |  |  |  | TP53 mut (R158L, 0,77%) | TP53 | R158L | 0.77 | NA |
| 1713 | pre-CRT | PASS | Yes | KRAS mut (G12C, 0,14%) | KRAS | G12C | 0.14 | NA |
|  | NA | FAIL | No | missing sample in inivata list | NA | NA | NA | NA |
|  | PD | PASS | Yes | KRAS mut (G12C, 0,50%) | KRAS | G12C | 0.5 | NA |
|  |  |  |  | TP53 mut (G334V, 0,61%) | TP53 | G334V | 0.61 | NA |
| 1925 | pre-CRT | PASS | Yes | KRAS mut (G12C, 1%) | KRAS | G12C | 1 | NA |
